# Students'psychological patterns and academic performance in public secondary schools in Kazo District, Uganda: a quantitative analysis

**DOI:** 10.1186/s40359-025-03187-w

**Published:** 2025-08-14

**Authors:** Lucy Aja, Innocent Sunday Odhine, Tukur Muhammad, Mohammad Lubega

**Affiliations:** 1 Department of Science Education, Faculty of Education, Kampala International University, Western Campus, Ishaka, Bushenyi Uganda; 2 Department of Foundation, Faculty of Education, Kampala International University, Western Campus, Ishaka, Bushenyi Uganda

**Keywords:** Academic performance, Public secondary schools, Quantitative analysis, Students'psychological patterns

## Abstract

**Background:**

This study investigates students’ psychological patterns and academic performance in public secondary schools in Kazo district, Uganda.

**Methodology:**

This study employed a cross-sectional research design and a quantitative methodology. The population comprised 697 secondary school students in Kazo District, with a sample size of 254 selected using Yamane’s technique. A simple random selection method was employed to pick the students who took part in the study. The data gathering instrument received validation from five specialists in educational administration and management. The research produced a reliability index of 0.842 for the students' questionnaire, calculated using Cronbach’s alpha. A self-administered questionnaire gathered quantitative data for the study, which was analyzed employing descriptive statistics.

**Findings:**

The results indicate that students in Kazo exhibit strong self-efficacy and confidence in their academic capabilities, which contributes positively to their motivation and engagement in learning, but there is a need to look at time management and pressure from academics to foster performance. The results also reveal that students have strong intrinsic motivation, which is linked to happiness in learning, and the importance of education to achieve future goals, whereas extrinsic factors such as parental or teacher supervision motivate the students. Finally, the study shows that there is a need for an effective support system to reduce academic stress and improve student academic performance.

**Conclusion:**

The integration of strong self-efficacy and intrinsic motivation among students positively influences their academic performance, whereas challenges such as anxiety, time management, and external pressures underscore the need for supportive resources.

**Recommendations:**

On the basis of the research findings, secondary schools should organize and implement workshops that focus on time management and study skills, which provide access to academic counseling for students facing challenges, fostering a positive learning environment that requires promoting intrinsic motivation through interactive teaching methods.

**Supplementary Information:**

The online version contains supplementary material available at 10.1186/s40359-025-03187-w.

## Introduction

The relationship between students'psychological patterns and academic success remains a challenge within the global educational and psychological research communities. There are recent studies that shows that psychological traits such as motivation, self-esteem, and emotional resilience are strongly connected to academic achievement, while negative states like anxiety, depression, and stress negatively impact students’ academic performance [20]. These relationships are often influenced by socio-economic, cultural, and systemic factors that differ across different countries globally. Similarly, global differences in socio-economic status remain a challenge, especially for the marginalized groups especially those facing poverty, inadequate educational resources, and pressure from the society all of which increase student’s psychological pattern and hinder academic performance [30]. From a global perspective, the relationship between psychological patterns and academic performance is complex and is been affected by cultural, socio-economic, and systemic factors that demand holistic strategies.

Uganda's educational framework has experienced many changes in recent decades, especially after the implementation of universal primary education (UPE) in 1997 and universal secondary education (USE) in 2007. According to Agyei, Annim, Acquah, et al. [1], policy changes intended to enhance educational access have also underscored inequities in student academic achievement. Multiple factors influence students'psychological patterns, including socioeconomic status, family background, and educational environment [14, 34]. Comprehending these psychological patterns is essential, as they influence students'academic motivation, self-efficacy, and, consequently, performance [4, 16]. Moreover, the impact of community support and engagement must not be overlooked, as community engagement frequently cultivates a nurturing educational atmosphere that can increase students'motivation and self-efficacy. Parents and local leaders who prioritize education can offer support and resources, hence enhancing students'psychological patterns [35]. Moreover, the quality of teacher-student connections profoundly influences students'academic engagement and emotional resilience [23].

Educators utilizing inclusive and supportive teaching methods in Uganda's educational framework have experienced many cases of negative familial situations, which promote a sense of belonging and competence in students [54]. Furthermore, the influence of cultural beliefs and practices significantly affects students'attitudes toward education [39]. According to Kuteesa, Akpuokwe, & Udeh [22], conventional perspectives on gender roles and educational achievement often generate inequities in motivation and access, particularly impacting girls'education. Addressing these cultural factors is important for establishing an educational atmosphere conducive to the success of all students. A thorough comprehension of the psychological patterns necessitates acknowledging individual factors as well as analyzing the interaction of societal structures that affect education. Successfully addressing these interrelated challenges can result in enhanced educational results and increased equity in academic performance among various student demographics [37].

Historically, Uganda's education system has faced various problems, including colonial legacies, political instability, and resource limitations. The emphasis on quantitative growth under UPE and USE has augmented enrollment but frequently undermined educational quality [31]. Kazo District exemplifies this tendency, as schools encounter challenges such as overcrowded classrooms, insufficient teaching resources, and disparate degrees of teacher preparation [24]. Moreover, societal perceptions of education, especially concerning gender, influence students'academic involvement and achievement [55]. Additionally, deficiencies in infrastructure have considerably obstructed the educational experience in Kazo District. Numerous schools function with inadequate infrastructure, sanitation amenities, and access to educational resources, which impedes the learning process and exacerbates students’ health problems. This infrastructural shortcoming is most obvious in rural regions, where the government has always faced challenges in distributing resources fairly. As a result, this fosters an environment in which students are less inclined to excel academically, owing to the unmet fundamental demands for safety and comfort inside the educational setting.

The ramifications of teacher quality and training are significant, and a significant number of instructors in the Kazo District lack sufficient professional development opportunities, leading staff to be potentially unprepared to meet the varied learning requirements of their students. The absence of training may result in inefficient pedagogical approaches that do not engage students or accommodate diverse skill levels [18]. The consequences are substantial, as students exhibit diminished academic performance and cultivate adverse attitudes toward school, potentially perpetuating cycles of poverty and marginalization in their communities. The relevance of the curriculum presents a considerable challenge. The national curriculum frequently fails to meet the needs and goals of local communities, resulting in student disinterest and disengagement. This disconnection exacerbates the challenges of educational quality and relevance, rendering students inadequately prepared for advanced education or the labor market [32]. It is essential to acknowledge that these difficulties are not isolated but rather interlinked within the wider socioeconomic and political framework of Uganda. Therefore, according to Buabeng & Amo-Darko. [6], addressing these issues requires a comprehensive strategy that considers the interplay of multiple elements, including economic development, community engagement, and policy reforms aimed at improving both access to and the quality of education. Uganda may achieve a fairer educational landscape that genuinely benefits all students only by addressing these fundamental obstacles.

This research is based on several psychological and pedagogical perspectives. Bandura's social cognitive theory highlights the significance of observational learning, imitation, and modeling in behavior, elucidating students'academic motivation and self-perceptions [3]. Observational learning is especially pertinent in Uganda, as students acquire knowledge not only via formal schooling but also by observing the behaviors and attitudes of peers, educators, and family members. This modeling process can profoundly affect their ambitions and perceived skills, hence influencing their learning strategies and resilience in overcoming obstacles. For example, according to Zimmerman [60], students who witness their classmates being commended for academic accomplishments may be inclined to establish loftier academic objectives, illustrating the impact of social effects on motivation. Self-determination theory asserts that intrinsic motivation profoundly impacts academic performance [8]. This approach emphasizes the importance of autonomy, competence, and relatedness as fundamental elements in cultivating students'intrinsic motivation. The Ugandan education system, which frequently prioritizes extrinsic rewards such as grades and parental expectations, faces the difficulty of fostering an environment in which students possess a sense of ownership over their learning. Students who exercise autonomy in their educational decisions, such as choosing projects aligned with their interests, tend to engage more profoundly and persistently with the curriculum. Studies have demonstrated that cultivating intrinsic motivation can enhance learning results, increase persistence, and promote a more favorable attitude toward educational endeavors [45].

Vygotsky’s social growth theory [56] emphasizes the importance of social interaction and the cultural context in cognitive growth and learning, especially within a communal society such as Uganda. Vygotsky asserted that cognitive processes are intrinsically linked to social interactions, which suggests that collaborative learning environments might improve students'understanding and retention of knowledge. In Uganda, where community and familial connections are robust, utilizing this social environment can be essential to education. Collaborative endeavors, peer tutoring, and group projects offer important opportunities for students to participate in substantive discussions, exchange viewpoints, and develop critical thinking abilities. Social engagement corresponds with the cultural subtleties of Ugandan society and can enhance the educational experience, promoting both academic achievement and social unity. Integrating these psychological and educational theories offers a complete framework for comprehending the elements that affect students'academic motivation and performance in Uganda. The study aims to clarify the relationships among social factors, intrinsic motivation, and the cultural context to improve educational achievements and address existing imbalances within the system. This theoretical perspective highlights that education in Uganda should focus not only on accessibility but also on enhancing the quality of the learning experience, ensuring that it is pertinent, engaging, and accommodating each student's distinct context and needs.

The concept of psychological patterns includes multiple facets, such as motivation, resilience, anxiety, and self-regulation. Motivation may be intrinsic or extrinsic, affecting students'involvement and perseverance in their academic pursuits [45]. Intrinsic motivation originates from authentic interest and pleasure in the learning experience, cultivating profound engagement with the content. This form of motivation is frequently associated with improved academic performance, as students who have personal significance in their studies are more inclined to engage actively and maintain their efforts over time. In contrast, extrinsic motivation, fuelled by external rewards or demands such as grades, parental expectations, or competitiveness, may result in more superficial engagement with learning. Comprehending the equilibrium between different motivational types is essential in educational contexts, especially in regions such as Uganda, where external influences may prevail, possibly compromising students'long-term interests and involvement [38].

According to Kalaivani [21], resilience denotes the capacity to recover from adversity, which is essential for academic achievement in demanding contexts. In districts such as Kazo District, where students face considerable socioeconomic difficulties, cultivating resilience is essential. Resilient students frequently demonstrate adaptive coping mechanisms, allowing them to overcome challenges such as limited resources and inadequate support systems [42]. Fostering resilience within educational systems can enhance students'ability to overcome challenges and sustain their academic progress [49]. Educational institutions and communities that cultivate resilient mindsets can establish supportive atmospheres that motivate students to perceive challenges as chances for development rather than as insurmountable obstacles [59]. Academic anxiety negatively impacts performance, resulting in a cycle of underachievement [11]. Anxiety in many students may present as fear of failure, test anxiety, or overall stress associated with academic pressures. This elevated emotional condition might hinder cognitive ability, leading to concentration issues, suboptimal decision-making, and thus diminished performance.

Academic worry frequently engenders a self-fulfilling prophecy in which the expectation of failure results in diminished effort and perseverance, hence exacerbating underachievement [13]. The implementation of school-based therapies to address anxiety, such as mindfulness training or cognitive-behavioral approaches, is important for fostering an educational climate that promotes learning and development. Self-regulation pertains to students'capacity to control their emotions, ideas, and behaviors in pursuit of their academic objectives [5]. Self-regulated learners excel in establishing objectives, tracking their advancement, and modifying their approaches as necessary. This skill is especially important in situations where pupils must manage external distractions and emotional difficulties. Instructing self-regulation techniques, including time management, goal setting, and reflective practices, can enable students to assume control over their learning processes. Furthermore, cultivating self-regulation can improve students'capacity to manage stressors, hence promoting increased academic engagement and performance [50].

These psychological patterns are important in formulating effective educational techniques that comprehensively satisfy students'requirements. By cultivating motivation, resilience, emotional regulation, and self-regulation within the educational framework, especially in challenging contexts such as Uganda, educators can more effectively assist students in their academic pursuits and overall personal development, resulting in transformative outcomes in their lives [2]. This holistic strategy can ultimately mitigate inequities in educational achievement and enable students to fulfill their potential in a swiftly evolving world. This study is paramount for educational stakeholders, such as policymakers, educators, and community leaders. Comprehending the psychological factors influencing academic achievement might guide specific interventions aimed at enhancing student results. By recognizing the distinct psychological challenges encountered by students, educators can create customized support systems, improve instructional methods, and cultivate a more favorable learning atmosphere. Moreover, policymakers can leverage these findings to develop policies that address wider socioeconomic variables affecting education in Kazo District.

Rectifying this deficiency is crucial for guiding initiatives designed to improve educational results in the district. Students'low self-esteem can substantially affect their academic performance. Studies demonstrated that self-esteem affects students'perceptions of their capabilities, thereby influencing their motivation to participate in academic activities [33]. In Kazo District, socioeconomic limitations and a scarcity of role models may intensify students’ feelings of inadequacy. Such emotions may foster a fixed mindset, wherein students perceive their abilities as immutable, hindering their readiness to confront difficulties and risk-taking in their educational pursuits [29]. This significantly increases anxiety, especially within academic settings, and affects students'educational experiences. The apprehension of unfavorable assessment or the compulsion to excel can result in heightened anxiety, which undermines students'capacity to focus and participate meaningfully in their academic pursuits. In Kazo District, elevated educational expectations stemming from cultural pressures can lead to a significant incidence of academic anxiety, hindering effective performance.

Interventions that provide students with coping mechanisms, including stress management techniques and relaxation exercises, are essential for alleviating anxiety and increasing their concentration on academic activities [41]. The absence of motivation, shaped by internal and extrinsic variables, exacerbates the educational environment in Kazo District. Numerous students may interact with their studies without interest or relevance, and this disengagement may stem from culturally specific obstacles, such as a curriculum that does not resonate with local circumstances or interests. Programs that advocate for culturally relevant pedagogy may substantially augment students'intrinsic motivation, as they recognize the significance and relevance of their learning. Moreover, improving educators'competencies to develop captivating and pertinent learning experiences can significantly contribute to rekindling students'enthusiasm for education [12]. The existing lack of empirical studies examining the relationships between these psychological characteristics and academic achievement in the Kazo District highlights an urgent need for investigation. Such investigations may yield significant insights into the impact of psychological patterns on educational achievements and pinpoint specific areas where interventions could be most efficacious.

The ramifications of this study permeate several levels of the educational background, and educators who identify psychological tendencies such as low self-esteem, anxiety, and lack of motivation can apply evidence-based approaches that address the emotional and psychological needs of their students [43]. Integrating social-emotional learning (SEL) into the curriculum enables educators to provide students with essential skills for emotional regulation and resilience development, and to foster a constructive attitude toward learning [10]. Programs that instruct on coping mechanisms for anxiety or techniques to promote self-regulation may empower students intellectually and personally, resulting in enhanced overall well-being [17]. Furthermore, educators can cultivate a supportive classroom environment that prioritizes growth mindset concepts, motivates students to accept challenges, and perceives mistakes as learning opportunities [19]. Training teachers to identify and address the individual needs of students effectively cultivates an atmosphere in which students feel appreciated and comprehended, which is important for inspiring their academic engagement [53].

Thus, customized support systems can markedly improve academic achievement, as students build confidence in their talents and foster favorable connections with their educational experiences.

The insights derived from this study are essential for policymakers in formulating educational policies that address the actual circumstances encountered by students in Kazo District. Addressing broader socioeconomic elements affecting education, including poverty, resource accessibility, and community involvement through legislative efforts, can establish an educational framework that promotes academic success. Policies that increase funding for schools in economically disadvantaged regions might enhance infrastructure, educational resources, and technological access, thereby mitigating certain gaps that intensify psychological obstacles to learning [28]. Thus, community outreach initiatives aimed at engaging parents and local leaders in the educational process can develop partnerships that enhance student learning experiences.

This study establishes a framework for stakeholders to apply evidence-based solutions that address the psychological aspects of learning by connecting educational research with practical applications. Enhancing collaboration among educators, policymakers, and community leaders establishes a robust foundation for fostering an educational climate in Kazo District that prioritizes the psychological well-being of students as important to their academic achievement. By doing so, educational stakeholders may improve student results and create a more equitable educational environment that benefits entire communities [57]. These research studies have the potential to revolutionize the educational experience in Kazo District and establish a foundation for sustainable development and lifetime learning opportunities for all students. Despite the initiatives to increase educational access in Uganda, considerable discrepancies in academic performance persist, especially among students in Kazo District, Uganda. Initial findings indicate that adolescents exhibit diverse psychological tendencies which may influence their academic difficulties. Subpar performance is frequently ascribed to variables such as diminished self-esteem, increased anxiety, and insufficient motivation. Nonetheless, there is a paucity of empirical research examining the correlation between these psychological traits and scholastic success in this area.

By documenting the intricate experiences of students in this location, researchers can formulate focused solutions that address the fundamental reasons for educational problems, thereby facilitating substantial enhancements in academic performance [51]. Comprehending and treating the psychological factors affecting students'academic achievement in Kazo District is crucial for cultivating a more equal and efficient educational environment. These initiatives seek to improve academic performance while simultaneously fostering students'general well-being and self-efficacy, equipping them with future challenges both academically and beyond. The gap from studies reveals that there isn't any real-world research that looks at how students'psychological patterns, like anxiety, motivation, self-efficacy, and academic achievement, connect with one another in public secondary schools in Kazo District, Uganda. People generally agree that these psychological factors have an effect on educational outcomes, but there isn't a lot of specific data and evidence-based insights that are specific to this region. Addressing these psychological factors through focused interventions will be essential for closing the present gaps in educational success and enhancing the overall educational environment in Uganda. This study considers it important to examine the correlation between students'psychological patterns and academic achievement in public secondary schools in the Kazo area, Uganda.

### Research objective

1. To evaluate the relationship between students'self-efficacy and their academic performance in secondary schools in Kazo District
2. To assess the impact of motivation, including intrinsic and extrinsic factors, on students’ academic performance in Kazo District
3. To identify the influence of anxiety and stress on students'academic performance in secondary schools in Kazo District.

### Research questions

1. What is the relationship between students'self-efficacy and their academic performance in secondary schools in Kazo District?
2. How does motivation, including intrinsic and extrinsic factors, impact students’ academic performance in the Kazo District?
3. How do anxiety and stress influence students'academic performance in secondary schools in Kazo District?

## Methodology

The methodology of the study is described below:

### Research design and research approach

The study employed a cross-sectional research design. This study, which was conducted in public secondary schools in Kazo District, uses a cross-sectional research design, which makes it easier to fully comprehend the direct relationships between students'psychological patterns and academic performance in public secondary schools in Kazo District, Uganda. This research design effectively identifies the study's objectives by allowing researchers to discern critical issues that may be addressed to improve educational outcomes. The study utilized a quantitative research approach. The examination of the relationship between students'psychological patterns and academic performance in public secondary schools in Kazo district, Uganda, is a good fit for quantitative research methodology. It facilitates objective assessment, enables comprehensive statistical analysis, and produces clear, widely applicable results that can inform educational practices and policies, ultimately improving regional educational outcomes.

### Target population, sample size, and sampling techniques

The target population comprised 697 secondary school students in Kazo District during the 2024/2025 academic year (District Education Officer Reports, 2024). A sample size of 254 was derived from a total population of 697 individuals using Yamane’s technique. A simple random selection method was employed to choose students who took part in the study.

### Instrument for data collection

The instrument for data collection was a self-developed questionnaire, and it was not previously published. The full version of the questionnaire has been included as supplementary material.

### Validity of the instruments for data collection

Five specialists in educational administration and management evaluated the data gathering instrument. The questionnaire items were evaluated for consistency, relevance, and clarity regarding the study objectives. Subsequently, the items were evaluated to confirm their alignment with the study's objectives, and the revisions provided by the experts were incorporated into the final version of the data collection instrument.

### Reliability of the instruments

A pilot study with 50 respondents was done to evaluate the reliability of the questionnaire. Their responses were examined using SPSS version 27. The construct's reliability was assessed using a benchmark of α = 0.70. The investigation, utilizing Cronbach's alpha, produced a reliability index of 0.842 for the students'questionnaire. The analysis surpassed the criterion of 0.70, signifying that the questionnaire was credible.

### Methods of data collection

A self-administered questionnaire was created and employed to gather quantitative data for the research investigation. Employing a questionnaire to gather quantitative data for investigating students'psychological patterns and academic performance in public secondary schools in Kazo district, Uganda, is a judicious and effective choice. Moreover, it is cost-effective and easy to analyze, offering standardization, efficiency, quantitative data, and the potential for extensive research. Due to these advantages, questionnaires serve as an effective method for understanding the factors affecting regional educational achievements.

### Method of data analysis

The quantitative data were analyzed via descriptive statistics. When analyzing quantitative data for the study of students'psychological patterns and academic performance in public secondary schools in the Kazo district, Uganda, descriptive statistics provide a straightforward, effective, and perceptive method of identifying trends and patterns. This strategy enhances educational practices and outcomes by promoting data-driven decision-making, facilitating efficient communication of findings, and providing a framework for more complex investigations.

## Results

**Fig. 1:**
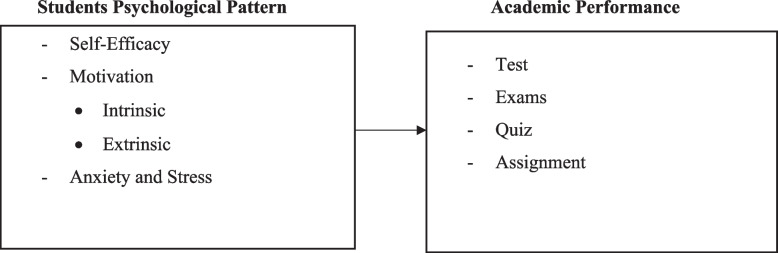
Shows the conceptual model of students'psychological patterns and academic performance in public secondary schools in Kazo District, Uganda

The following is the presentation of the results and discussion of the findings:

Figure 1 shows that, among 254 students whose questionnaires were sent out to respondents, 228 questionnaires were returned, which represented a response rate of 90.1%.

**Fig. 2:**
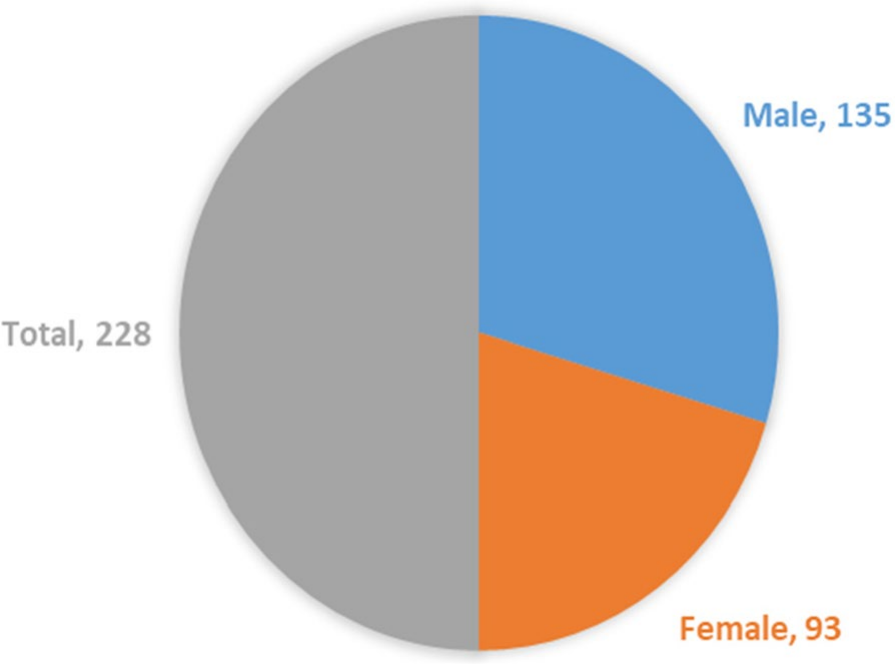
Participant demography

Figure 2 shows that 135 (59%) participants were male, whereas the remaining 93 (41%) were female, for 228 students.

### Research question one: what is the relationship between students'self-efficacy and their academic performance in secondary schools in Kazo District?

Table 1 shows the responses to ten self-efficacy items on a 5-point Likert scale, with the average mean scores indicating the overall sentiment toward students'confidence and perceived effectiveness in academic performance. Item 1 has a high mean score of 4.43, suggesting that students strongly believe they can achieve good grades. Item 2 follows with a mean score of 4.26, indicating confidence in understanding new concepts. Item 3 has a mean of 4.04, reflecting mixed feelings about completing assignments on time. Items 4 and 7 have means of 4.10 and 4.24, respectively, signifying that students set goals and perceive their efforts as contributing to success. Item 5 has a lower mean score of 3.97, highlighting challenges faced with difficult materials. Items 6 and 8 show high mean scores of 4.18 and 4.25, indicating confidence in exam preparation and support from teachers. Item 9 has a mean of 3.94, denoting difficulties with time management, while item 10 has the lowest mean of 3.69, reflecting struggles with handling academic pressure.

**Table 1:** To evaluate the relationship between students'self-efficacy and their academic performance in secondary schools in Kazo District

S/N	QUESTIONS	SD	D	N	A	SA	Mean
1	I believe I can achieve good grades in my classes	01	00	20	86	122	4.4323
2	I have confidence in my ability to grasp new concepts	00	04	14	129	81	4.2588
3	I feel capable of completing my assignments on time	01	15	31	96	73	4.0417
4	I often set high academic goals for myself	03	19	18	75	84	4.0955
5	I can overcome challenges when studying difficult materials	05	17	41	79	84	3.9735
6	I am confident in my ability to prepare for exams efficiently	07	07	22	89	96	4.1765
7	I believe that my academic efforts have led to success	05	07	25	85	111	4.2446
8	I feel well supported by my teachers to excel academically	01	10	21	90	102	4.2589
9	I can manage my study time effectively	06	15	40	79	76	3.9444
10	I can handle academic pressure without losing focus	16	40	32	82	81	3.6853

Source: Primary data

### Research question two: how does motivation, including intrinsic and extrinsic factors, impact students’ academic performance in the Kazo District?

Table 2 presents responses to ten items measuring students’ motivation to learn and achieve academically on a 5-point Likert scale. The analysis grouped the items into intrinsic, extrinsic, and mixed motivation factors. For intrinsic motivation, item 1 has a high mean score of 4.36, indicating that students generally enjoy learning new things. Item 2 also reflects strong intrinsic motivation with a mean of 4.04, showing that students are motivated by achieving high grades for personal satisfaction. Item 6 has a mean score of 4.28, suggesting that students see education as a pathway to future achievement, and item 8’s mean of 4.29 indicates that students believe academic success will lead to better opportunities. In terms of extrinsic motivation, item 3 has a mean of 3.91, highlighting that recognition encourages students to work harder, while item 5 has a mean of 4.22, emphasizing the motivating influence of parental or teacher supervision. Peer support, represented by item 7, has a mean score of 4.30, indicating a positive impact of peer relationships on motivation. For mixed motivation, item 4 has a mean of 4.18, showing that active class participation is motivated by both interest and external factors. Items 9 and 10 show lower scores of 3.85 and 3.98, respectively, indicating that external rewards and class competition serve as moderate motivators. Overall, the results suggest that students exhibit strong intrinsic motivation, supported by extrinsic factors, with intrinsic motivation playing a central role in fostering academic engagement.

**Table 2:** To assess the impact of motivation, including intrinsic and extrinsic factors, on students’ academic performance in Kazo District

S/N	QUESTIONS	SD	D	N	A	SA	Mean
1	I enjoy learning new things in school	01	02	39	60	129	4.3593
2	I am motivated to achieve high grades for personal satisfaction	00	06	33	137	55	4.0433
3	The recognition I receive for my academic achievements encourages me to study harder	10	10	44	108	72	3.9098
4	I participate actively in class because I am interested in the subject matter	03	15	27	88	111	4.1844
5	I study harder when I know my parents or teachers are watching	04	04	32	91	104	4.2213
6	I value education as a way to achieve my future goals	00	03	26	103	96	4.2807
7	I feel supported by my peers to perform well academically	03	04	37	48	116	4.2981
8	I believe my academic success will lead to better opportunities in the future	02	15	20	69	122	4.2895
9	I would study harder if rewards were given for good performance	11	21	25	97	66	3.8455
10	I think competition among my classmates drives me to do my best	11	11	31	93	82	3.9825

Source: Primary data

### Research question 3: how do anxiety and stress influence students'academic performance in secondary schools in Kazo District?

Table 3 summarizes students'experiences related to anxiety and stress in connection with their academic performance, measured on a 5-point Likert scale. The average scores reflect the extent of students'feelings regarding different stressors. Item 1,"I often feel anxious about performing well on exams,"has a mean score of 3.95, indicating that many students experience academic exam anxiety. Item 2 shows a lower mean of 2.89, suggesting that stress negatively affects concentration. Regarding how anxiety impacts performance, item 3 has a mean of 3.31, showing mixed feelings among students about the effect of grade-related anxiety. Concerns about disappointing parents and teachers yield a mean score of 3.46 in item 4. Excessive academic workload is reported as a stressor, with a mean of 2.80. Physical stress symptoms like headaches are less commonly reported, with a mean score of 2.23. Sleep difficulties are moderately reported, with a mean of 2.89. Anxiety's effect on class participation is reflected in a mean of 3.33, indicating some students feel it hinders engagement. The use of coping mechanisms shows variability, with a mean score of 3.20. Lastly, students'belief in the benefits of addressing stress has a mean of 3.81.

**Table 3:** To identify the influence of anxiety and stress on students'academic performance in secondary schools in Kazo District

S/N	QUESTIONS	SD	D	N	A	SA	Mean
1	I often feel anxious about performing well on exams	11	17	42	60	98	3.9518
2	I find it hard to concentrate on my studies because of stress	57	36	38	68	28	2.8855
3	My anxiety regarding grades affects my ability to perform during tests	07	44	69	71	27	3.3073
4	I worry about disappointing my teachers and parents with my academic performance	14	34	42	94	34	3.4587
5	I frequently feel overwhelmed by the academic workload	23	74	69	29	24	2.8037
6	I experience physical symptoms, such as headaches, when I think about exams	78	60	32	26	14	2.2286
7	I have difficulty sleeping due to concerns about my academic performance	38	57	40	53	28	2.8889
8	I feel that my anxiety impacts my class participation	12	51	52	50	47	3.3255
9	I use coping mechanisms, like deep breathing, to manage academic stress	24	56	56	75	39	3.1960
10	I believe that addressing my anxiety would help improve my grades	10	18	45	69	71	3.8122

Source: Primary data

## Discussion of findings

### What is the relationship between students'self-efficacy and their academic performance in secondary schools in Kazo District

**Fig. 3:**
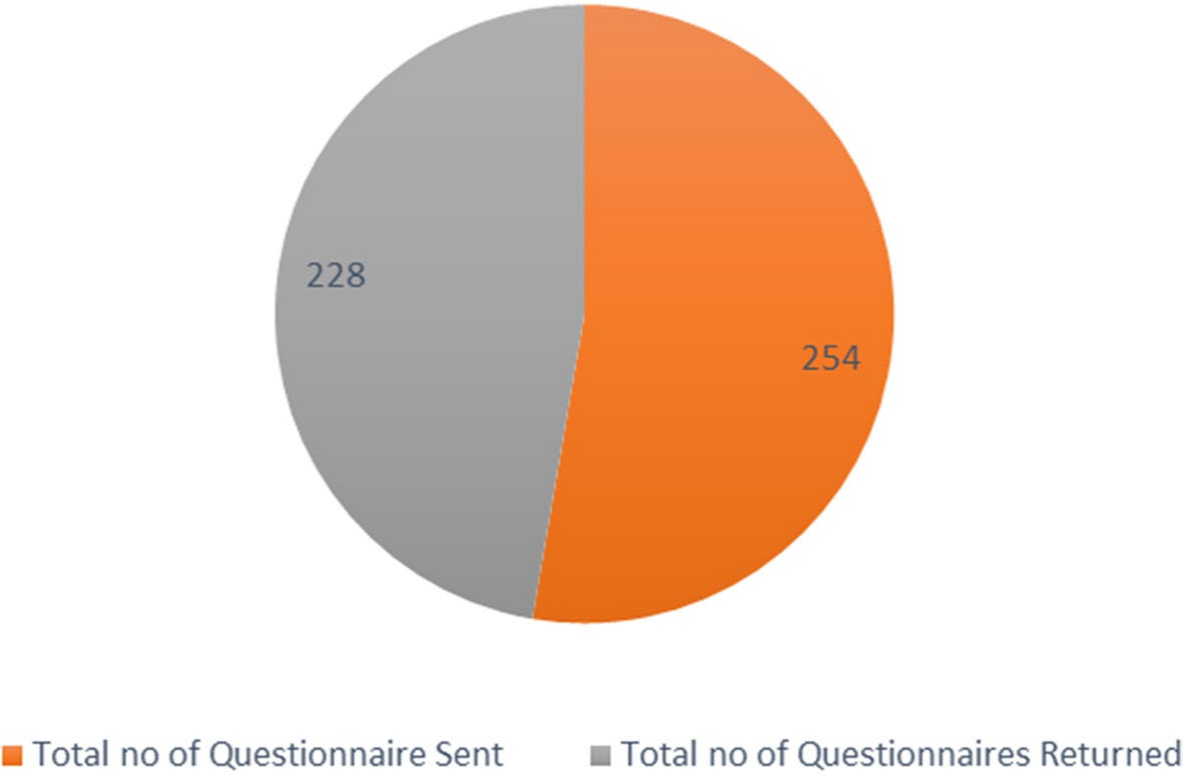
Student questionnaire response rate

The results reveal that students in Kazo District generally exhibit strong self-efficacy, especially in their confidence to achieve good grades and set goals, supporting Bandura's (1997) theory that higher self-efficacy fosters setting challenging goals and persistence. Confidence in grasping new concepts aligns with research by Schunk [47] and Liu, Du, & Lu [25], emphasizing that belief in learning abilities encourages deeper engagement. However, lower scores in time management and handling pressure indicate areas where students face difficulties, which could undermine their academic success. The challenges in managing time and stress corroborate findings by Lovin & Bernardeau-Moreau [26] and Pascoe et al. [40], highlighting the need for targeted support to improve these skills. Similarly, difficulties with difficult materials suggest a need for additional academic support, as noted by Basileo et al. [4]. External support from teachers appears to positively reinforce self-efficacy, consistent with research by Tao et al. [53]. Overall, while students demonstrate confidence and motivation, addressing time management and stress management could further enhance their academic performance, emphasizing the importance of providing adequate resources and support systems. The high average scores across these different items suggest a positive perception of self-efficacy, based on theories of educational psychology, it is strongly related with higher academic performance. Students who have confidence in their capabilities are more likely to engage actively in learning, persist through challenges, and achieve academical performance. In addition, the responses from the students indicate that most students feel supported by teachers and are confident in their ability to set goals and overcome challenges, reinforcing a positive self-efficacy mindset conducive to academic performance. In conclusion, based on the results, it strongly suggest that there is a positive relationship between students'self-efficacy and their academic performance in Kazo District secondary schools Fig. 3.

### How does motivation, including intrinsic and extrinsic factors, impact students’ academic performance in the Kazo District?

The findings reveal that intrinsic motivation significantly influences students’ academic behavior, particularly in their enjoyment of learning and pursuit of long-term goals. The high scores for items related to intrinsic motivation align with research by Onyekwere, Okoro, & Unamba [38] and Ryan and Deci [44], which underscore that internal interest and perceived relevance of education foster sustained engagement and achievement. The data also emphasize that external motivators such as recognition, supervision, and peer support serve as significant but supplementary factors, corroborating prior research that external validation can motivate students but may not be the primary driver of effort [9, 53]. The moderate influence of competition and external rewards suggests that while these factors can enhance motivation temporarily, intrinsic satisfaction and personal relevance are more powerful and sustainable motivators, as supported by studies from Nayir [36] and Ma & Chen [27]. Overall, the findings suggest that fostering intrinsic interest and linking education to students’ future goals could be more effective strategies for enhancing academic motivation and performance in Kazo District. These findings reveal that both intrinsic motivations, such as enjoyment and valuing education, and extrinsic motivations, like recognition and competition, positively influence students'academic performance. Students in Kazo District seem to be motivated by a mix of internal interests and external rewards, which together could boost their engagement, effort, and improve their academic performance.

### How do anxiety and stress influence students'academic performance in secondary schools in Kazo District?

The results indicate that a significant portion of students in Kazo District experience exam-related anxiety, which can impact their focus and class participation. These findings concur with Chakraborty's [7] research identifying exam anxiety as a widespread issue that affects mental health and academic performance. The moderate to low scores for physical symptoms and sleep issues suggest some resilience among students, possibly due to effective coping strategies, as highlighted by Sameer & Naved [46]. The impact of academic workload and fear of disappointing significant others corroborates existing literature on the detrimental effects of excessive demands and social pressures on student well-being, as supported by Xia et al. [58] and Suman [52]. The findings also emphasize that while some students employ coping mechanisms to manage stress, others do not, aligning with research by Handayani et al. [15], which emphasizes the importance of effective stress management strategies. Overall, these findings suggest that addressing academic-related anxiety and stress through support systems and coping skills training could improve students'concentration, participation, and overall academic performance in the Kazo District. The results show that while many students feel anxious and overwhelmed, they also recognize that managing their stress could improve their academic performance. In conclusion, anxiety and stress influence students’ ability to perform academically, thereby affecting the concentration, physical health, and class participation of the students.

## Conclusion

In conclusion, the integration of strong self-efficacy and intrinsic motivation among students positively influences their academic performance, whereas challenges such as anxiety, time management, and external pressures underscore the need for supportive resources. Addressing these issues through effective coping strategies and supportive academic environments is essential to enhance student well-being and foster greater engagement in learning. Ultimately, prioritizing emotional resilience and intrinsic satisfaction will be key to improving overall academic outcomes.

### Recommendation

Based on the research findings, recommendations were made to increase student academic performance. Secondary schools should organize and implement workshops that focus on time management and study skills that providing access to academic counseling for students facing challenges, fostering a positive learning environment that requires promoting intrinsic motivation through interactive teaching methods that relate lessons to real-life applications, thereby recognizing student achievements to build confidence. Additionally, programs that are organized to teach students effective coping strategies should be developed to help manage academic stress, pressure, and anxiety. Finally, teachers should provide constructive and timely feedback on academic performance that will help students understand their strengths and areas for improvement to overcome challenges that will lead to improved academic performance.

### Theoretical implications of the findings

The study supports Bandura's (1997) theory of self-efficacy by showing that students'confidence has a direct connection to better grades. The results support earlier studies, like Schunk [48] and Liu et al. [26], which stress that having faith in one's ability to learn makes people more engaged and persistent. The results about motivation are in line with Ryan and Deci's [44] Self-Determination Theory. They show that intrinsic motivation, enjoyment, and personal relevance have a greater and longer-lasting influence on academic performance than external influences like rewards and recognition. Also, the effect of anxiety and stress on academic performance is in line with existing psychological models, which supports the idea that emotions are very important for cognitive functioning and academic success [58]. Finally, the study's results show how important it is to include psychological principles when trying to understand how students perform academically in school.

### Practical implications of the findings

The study's practical implications suggest that educators should help students feel more capable by giving them exercises that help them set goals. Intrinsic motivation, which makes learning relevant, should be emphasized, along with the goal set by the students to improve their academic performance. Schools should also hold workshops, seminars, and counselling sessions to help students deal with the stress and anxiety that comes with schoolwork.

### Limitations of the study

The study has several limitations that need to be mentioned. First, the study was done in secondary schools in Kazo District, Uganda. This may limit the results that can be applied to other districts, regions, and countries with different cultures and social backgrounds. Using a cross-sectional research design makes it harder for observing how these variables changes over time. The study also used quantitative methods, which are useful for finding patterns and relationships but don't fully show how students feel or the factors that affect their academic performance. These limitations point out areas where more research is needed to make the results in this field more reliable and useful.

### Suggestions for future research

This study pointed out a possible area for future research that should try to fix some of the limitations with the current study and learn more about the things that affect how well students do in school. The study suggested using a longitudinal research design to see how self-efficacy, motivation, and stress levels change over time and how they continue to affect academic performance. Using qualitative research methods like interviews can also help better understand how students feel, how they deal with problems, and the things around them that affect their academic performance. Also, experimental studies are needed to see if targeted strategies that were meant to help students improve their self-efficacy, motivation, and stress management are working. Including different regions and types of schools, both rural and urban, in the research will help find contextual factors that affect these psychological patterns.

## Supplementary Information

Supplementary Material 1.

## Data Availability

Data that support the findings of this study have been deposited in OSF repository, and it has a DOI number: 10.17605/OSF.IO/MWCA3.
