# Supplementary material for: Students'psychological patterns and academic performance in public secondary schools in Kazo District, Uganda: a quantitative analysis
Source: BMC Psychol. 2025 Aug 14;13:920. doi: 10.1186/s40359-025-03187-w (PMC12355781; doi:10.1186/s40359-025-03187-w)
Supplement: Supplementary file 1 — Supplementary Material 1. [file 40359_2025_3187_MOESM1_ESM.docx]

Researcher: Introductory Letter

Dear Participant's

We are researchers conducting research in students' psychological patterns and academic performance in public secondary schools in Kazo district, Uganda: a quantitative analysis. We would like to invite you to participate in a questionnaire that aims to capture the insights and experiences about the above topic. Your perspective is invaluable, and your responses will greatly contribute to a better understanding of current practices and potential areas for improvement within our schools.

The questionnaire will take approximately 10-15 minutes to complete. Please be assured that all responses will be kept confidential and used solely for academic purposes. Participation in this study is voluntary, and you may withdraw anytime.

Sincerely,


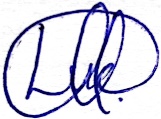


…………..

For researchers

**Section 1. Socio-demographic characteristics of the participants. Please tick according to your appropriate decision.**

**Gender:**

- 1. **Male**
  2. **Female**

**Section 2. To evaluate the relationship between students' self-efficacy and their academic performance in secondary schools in Kazo District**

| S/N | QUESTIONS | SD | D | N | A | SA |
| --- | --- | --- | --- | --- | --- | --- |
| 1 | I believe I can achieve good grades in my classes |  |  |  |  |  |
| 2 | I have confidence in my ability to grasp new concepts |  |  |  |  |  |
| 3 | I feel capable of completing my assignments on time |  |  |  |  |  |
| 4 | I often set high academic goals for myself. |  |  |  |  |  |
| 5 | I can overcome challenges when studying difficult materials |  |  |  |  |  |
| 6 | I am confident in my ability to prepare for exams efficiently |  |  |  |  |  |
| 7 | I believe that my academic efforts lead to success |  |  |  |  |  |
| 8 | I feel well supported by my teachers to excel academically |  |  |  |  |  |
| 9 | I can manage my study time effectively |  |  |  |  |  |
| 10 | I can handle academic pressure without losing focus. |  |  |  |  |  |

**Section 3. To assess the impact of motivation, including intrinsic and extrinsic factors, on students’ academic performance in Kazo District.**

| S/N | QUESTIONS | SD | D | N | A | SA |
| --- | --- | --- | --- | --- | --- | --- |
| 1 | I enjoy learning new things in school |  |  |  |  |  |
| 2 | I am motivated to achieve high grades for personal satisfaction |  |  |  |  |  |
| 3 | The recognition I receive for my academic achievements encourages me to study harder |  |  |  |  |  |
| 4 | I participate actively in class because I am interested in the subject matter |  |  |  |  |  |
| 5 | I study harder when I know my parents or teachers are watching |  |  |  |  |  |
| 6 | I value education as a way to achieve my future goals |  |  |  |  |  |
| 7 | I feel supported by my peers to perform well academically |  |  |  |  |  |
| 8 | I believe my academic success will lead to better opportunities in the future |  |  |  |  |  |
| 9 | I would study harder if rewards were given for good performance |  |  |  |  |  |
| 10 | I think competition among my classmates drives me to do my best. |  |  |  |  |  |

**Section 4. To identify the influence of anxiety and stress on students' academic performance in Kazo District secondary schools.**

| S/N | QUESTIONS | SD | D | N | A | SA |
| --- | --- | --- | --- | --- | --- | --- |
| 1 | I often feel anxious about performing well on exams |  |  |  |  |  |
| 2 | I find it hard to concentrate on my studies because of stress. |  |  |  |  |  |
| 3 | My Anxiety regarding grades affects my ability to perform during tests. |  |  |  |  |  |
| 4 | I worry about disappointing my teachers and parents with my academic performance. |  |  |  |  |  |
| 5 | I frequently feel overwhelmed by the academic workload |  |  |  |  |  |
| 6 | I experience physical symptoms, such as headaches, when I think about exams. |  |  |  |  |  |
| 7 | I have difficulty sleeping due to concerns about my academic performance |  |  |  |  |  |
| 8 | I feel that my anxiety impacts my class participation |  |  |  |  |  |
| 9 | I use coping mechanisms, like deep breathing, to manage academic stress |  |  |  |  |  |
| 10 | I believe that addressing my anxiety would help improve my grades |  |  |  |  |  |
